# Vaccine Hesitancy Is a Barrier to Achieving Equitable Herd Immunity Among Racial Minorities

**DOI:** 10.3389/fmed.2021.668299

**Published:** 2021-11-24

**Authors:** Philip Gerretsen, Julia Kim, Lena Quilty, Samantha Wells, Eric E. Brown, Branka Agic, Bruce G. Pollock, Ariel Graff-Guerrero

**Affiliations:** ^1^Centre for Addiction and Mental Health (CAMH) and Department of Psychiatry, Campbell Family Mental Health Research Institute, University of Toronto, Toronto, ON, Canada; ^2^Institute of Medical Science, School of Graduate Studies, University of Toronto, Toronto, ON, Canada; ^3^Dalla Lana School of Public Health, University of Toronto, Toronto, ON, Canada

**Keywords:** COVID-19, racial minorities, herd immunity, vaccine hesitancy, vaccine acceptance, 3C model

## Abstract

**Introduction:** Racial minority groups have been disproportionately affected by the 2019 novel coronavirus disease (COVID-19). Vaccine hesitancy may be a major barrier to achieving equitable herd immunity and must be addressed to reduce the excess morbidity and mortality of COVID-19 in disproportionately affected communities. This study aimed to determine if COVID-19 vaccine hesitancy, and its factors vaccine complacency and confidence, are more prominent among disproportionately affected racial minority groups.

**Methods:**We collected data from participants aged 18 years or older from the four most populous U.S. states, including New York, California, Florida, and Texas, and Canada. Data were collected using a web-based survey platform. Data are available at http://www.covid19-database.com.

**Results:**Data from 4,434 participants were included [mean (SD) age = 48.7 (17.2) and 50.4% women]. Vaccine hesitancy was higher in Black, Indigenous (Native American and Indigenous People of Canada, including First Nations, Inuit and Métis), and Latinx compared to White participants, while no difference was found between East Asian and White participants. The group differences in vaccine hesitancy for Indigenous and Black compared to White participants remained after controlling for sociodemographic factors. Determinants of vaccine complacency were equivalent between disproportionately affected racial groups and white participants. Vaccine confidence (i.e., trust in vaccine benefit) was generally lower in all racial groups compared to White participants. Differences in vaccine mistrust comparing Black and East Asian to White participants remained after controlling for sociodemographic factors.

**Discussion:**Disproportionately affected racial minorities may have higher vaccine hesitancy and lower confidence in COVID-19 vaccines. Public health and other relevant government services should address vaccine hesitancy among racial minorities using a culturally sensitive, community-centered approach to attain equitable herd immunity.

## Introduction

The 2019 novel coronavirus disease (COVID-19) has disproportionately affected racial minorities in the United States (U.S.) and Canada, resulting in higher rates of infection, hospitalization and death (1–5). Black, Indigenous, and Latinx (i.e., people of Latin American cultural or ethnic identity) populations have had ≥2.6 times higher case rates (2) and ≥3.3 times higher mortality rates than non-Latinx White individuals (6). To address these health disparities, the National Academies of Sciences, Engineering, and Medicine (NASEM) and the World Health Organization (WHO) have recommended prioritization of racial minorities who are socioeconomically and epidemiologically disadvantaged for COVID-19 vaccines (2, 7). To accomplish this in the U.S., indices are available, including the Social Vulnerability Index (8) and the Area Deprivation Index (9), to guide the equitable distribution of vaccines based on regional socioeconomic status.

Despite efforts to promote equitable distribution of vaccines, vaccine hesitancy is a likely barrier to achieving herd immunity and reducing the excess morbidity and mortality attributable to COVID-19. Some evidence suggests that Indigenous (Native American and Indigenous People of Canada, including First Nations, Inuit and Métis), Black, and Latinx individuals have higher rates of vaccine hesitancy; however, this research lacks information on the key underlying drivers (10–13). An effective framework for equitable vaccine allocation to disproportionately affected racial minorities must address vaccine hesitancy in these groups to ensure equitable herd immunity.

According to the WHO Strategic Advisory Group of Experts (SAGE), vaccine hesitancy emerges when individuals (1) *lack confidence* in the safety and effectiveness of the vaccine and the system recommending and providing it; (2) are *complacent*, in that they do not believe the vaccine-preventable disease is serious and vaccination is not necessarily required to prevent infection; and (3) perceive that access to the vaccine is *inconvenient*, uncomfortable or unaffordable (14). The present paper focuses on vaccine hesitancy and the determinants of vaccine complacency and confidence. Governments are tasked with the responsibility of ensuring vaccines are convenient [i.e., easily accessible, affordable, and delivered in a comfortable and culturally sensitive manner (14)].

In a large sample of people in the U.S. and Canada, this study aimed to determine whether there are differences in vaccine hesitancy, complacency, and confidence across the following racial/ethnic groups: Indigenous, Black, Latinx, East Asian and White. We hypothesized disproportionately affected racial minority groups would have higher COVID-19 vaccine hesitancy attributable to differences in the determinants of lower vaccine confidence compared to East Asian and White participants.

## Methods

We collected data from 4,434 participants aged 18 years or older from the four most populous U.S. states, including New York (*n* = 1001), California (*n* = 1001), Florida (*n* = 501), and Texas (*n* = 503) and from English-speaking Canada (*n* = 1936). Data are available at http://www.covid19-database.com. Data were collected at three time points, in May and July 2020 using a web-based survey. The survey was developed, pre-tested, and collected using a web-based platform *Dynata*, a global market research company. We placed a quota restriction on age to ensure that data from a representative sample of participants from the U.S. and Canada were collected. We aimed to include approximately an equal number of respondents from the following age ranges: 18–24, 25–34, 35–44, 45–54, 55–64, and 65+ years of age from each region. Responses were collected between May to August 2020. The study was approved by our institution's Research Ethics Board (REB). All participants provided informed consent prior to starting the survey.

Participants provided sociodemographic information and completed a battery of assessments to assess vaccine hesitancy and the determinants of vaccine complacency and confidence in relation to COVID-19. Participants were asked to select the racial or ethnic group that best describes them. Participants that selected the following categories were included for analysis: “Indigenous” (Native American, American Indian, First Nations, Inuit and Métis), “Black,” “Latinx” (Hispanic), “East Asian” (Chinese, Japanese, or Korean), and “White.” These categories were chosen based on the NIH guidelines for racial and ethnic categories and by Statistics Canada (15, 16). Not all racial groups were included in the analysis, including Arab/West Asian, Filipino, Southeast Asian, South Asian, and “Other groups.” Our study was a direct follow-up investigation of an editorial published in *JAMA* that discussed the prioritization of COVID-19 vaccinations in disproportionately affected racial minorities (1). As a result, we focused our investigation on these disproportionately affected groups, which included Indigenous, Black, and Latinx individuals. Participants' degree of vaccine hesitancy was assessed using a single-item that asked how likely they are to get vaccinated if a vaccine for COVID-19 becomes available. The answer option ranged from “1, Definitely” to “6, Definitely Not,” with a higher score representing greater hesitancy. Assessments of the determinants of vaccine complacency and confidence are presented in Table 1.

**Table 1:** Differences in sociodemographic and vaccine hesitancy, complacency, and confidence determinants between racial groups.

	**Indigenous**	**Black**	**Latinx**	**East Asian**	**White**	
	**(*N* = 48)**	**(*N* = 219)**	**(*N* = 338)**	**(*N* = 529)**	**(*N* = 3,300)**	
	**Mean (SD) or** ***N*** **(%)**					***t*** **(df) and** ***p*****-value**
Vaccine hesitancy score	3.1 (1.8)	3.5 (1.8)	2.6 (1.7)	2.4 (1.5)	2.2 (1.5)	*F*_(4, 4429)_ = 41.22, *p* <0.001^*^^1^, ^2^, ^3^
**Sociodemographic determinants**						
Age	43.4 (17.3)	40.3 (17.3)	40.4 (17.0)	39.8 (14.2)	51.6 (16.7)	*F*_(4, 4429)_ = 100.48, *p* <0.001^*^^5^, ^6^, ^7^, ^8^
Gender (man/woman^a^)	24 (51.1%)/23 (48.9%)	90 (41.3%)/128 (58.7%)	147 (43.6%)/190 (56.4%)	240 (45.5%)/287 (54.5%)	1,681 (51.1%)/1,606 (48.9%)	χ^2^(4) = 17.43, *p* = 0.002
Education (years)	15.0 (4.0)	14.0 (4.0)	14.0 (4.0)	16.3 (3.5)	15.0 (4.0)	*F*_(4, 4428)_ = 21.27, *p* <0.001^*^^4^, ^6^, ^7^
Religion (yes/no^a^)	22 (52.4%)/20 (47.6%)	160 (76.2%)/50 (23.8%)	250 (77.9%)/71 (22.1%)	215 (42.7%)/289 (57.3%)	2,133 (66.9%)/1,055 (33.1%)	χ^2^(4) = 153.90, *p* <0.001^*^^3^, ^8^
**Region**						
New York/California^a^	12 (25.0%)	105 (47.9%)	158 (46.7%)	228 (43.1%)	1,336 (40.5%)	–
Canada	31 (86.1%)	48 (21.9%)	26 (7.7%)	259 (49.0%)	1,316 (67.0%)	χ^2^(4) = 119.51, *p* <0.001^*^^6^, ^7^
Florida/Texas	5 (13.9%)	66 (30.1%)	154 (45.6%)	42 (7.9%)	648 (33.0%)	χ^2^(4) = 77.22, *p* <0.001^*^^3^, ^8^
**Population density**						
1,000 or less^a^	4 (8.3%)	6 (2.8%)	6 (1.8%)	5 (1.1%)	103 (3.2%)	–
1,000–29,999	7 (14.6%)	16 (7.5%)	27 (8.0%)	20 (4.2%)	396 (12.3%)	χ^2^(4) = 2.30, *p* = 0.681
30,000–99,999	13 (27.1%)	36 (16.9%)	54 (16.0%)	68 (14.4%)	515 (16.0%)	χ^2^(4) = 7.13, *p* = 0.129
100,000 or more	21 (43.8%)	133 (62.4%)	207 (61.2%)	378 (80.3%)	1,961 (60.8%)	χ^2^(4) = 19.74, *p* = 0.001^*^^4^
**Household income**						
< $20,000^a^	3 (6.5%)	39 (18.7%)	48 (15.0%)	24 (4.8%)	178 (5.7%)	–
$20,000–$59,999	19 (41.3%)	77 (37.0%)	116 (36.1%)	116 (23.4%)	770 (24.7%)	χ^2^(4) = 22.69, *p* <0.001^*^^2^, ^3^
$60,000–$99,999	12 (26.0%)	53 (25.4%)	90 (28.0%)	159 (32.1%)	896 (28.8%)	χ^2^(4) = 62.00, *p* <0.001^*^^6, 7^
$100,000–$139,999	9 (19.6%)	20 (9.6%)	34 (10.6%)	94 (18.9%)	597 (19.2%)	χ^2^(4) = 93.81, *p* <0.001^*^^6, 7^
$140,000 or more	3 (6.5%)	19 (9.1%)	33 (10.3%)	103 (20.8%)	675 (21.7%)	χ^2^(4) = 114.77, *p* <0.001^*^^6, 7^
**Employment status**						
Unemployed	8 (16.7%)	41 (18.7%)	52 (15.4%)	66 (12.9%)	358 (10.8%)	χ^2^(4) = 14.51, *p* = 0.006^*^^2^
Employed^a^	30 (62.5%)	112 (51.1%)	180 (53.3%)	352 (68.8%)	1,762 (53.4%)	–
Student	1 (2.1%)	27 (12.3%)	46 (13.6%)	51 (9.9%)	94 (2.8%)	χ^2^(4) = 102.93, *p* <0.001^*^^2, 3, 4^
Retired	8 (16.7%)	30 (13.7%)	42 (12.4%)	43 (8.4%)	921 (27.9%)	χ^2^(4) = 112.40, *p* <0.001^*^^6, 7, 8^
Healthcare worker (yes/no^a^)	5 (10.4%)/43 (89.6%)	50 (22.8%)/169 (77.2%)	56 (16.6%)/282 (83.4%)	91 (17.2%)/438 (82.8%)	379 (11.5%)/2,921 (88.5%)	χ^2^(4) = 37.47, *p* <0.001^*^^4, 6^
	**Mean (SD) or** ***N*** **(%)**					***t*** **(df) and** ***p*****-value**
**Political spectrum**						
Communism left wing or socialism	1 (2.1%)	10 (4.6%)	20 (5.9%)	18 (3.4%)	203 (6.2%)	χ^2^(4) = 14.68, *p* = 0.005^*^^8^
Liberal	14 (29.2%)	73 (33.3%)	103 (30.5%)	165 (31.2%)	945 (28.6%)	χ^2^(4)=3.71, p=0.447
Center^a^	16 (33.3%)	92 (42.0%)	142 (42.0%)	217 (41.0%)	1,067 (32.3%)	–
Conservative	15 (31.3%)	33 (15.1%)	70 (20.7%)	120 (22.7%)	1,025 (31.1%)	χ^2^(4) = 55.36, *p* <0.001^*^^6, 7, 8^
Fascism right wing or authoritarianism	2 (4.2%)	11 (5.0%)	3 (0.9%)	9 (1.7%)	60 (1.8%)	χ^2^(4) = 11.04, *p* = 0.026
Alcohol use (yes/no^a^)	23 (47.9%)/25 (52.1%)	119 (54.3%)/100 (45.7%)	189 (55.9%)/149 (44.1%)	277 (52.4%)/252 (47.6%)	2,267 (68.7%)/1,033 (31.3%)	χ^2^(4) = 86.09, *p* <0.001^*^^5, 6, 7, 8^
Cigarette use (yes/no^a^)	10 (20.8%)/38 (79.2%)	40 (18.3%)/179 (81.7%)	58 (17.2%)/280 (82.8%)	72 (13.6%)/457 (86.4%)	666 (20.2%)/2,634 (79.8%)	χ^2^(4) = 13.84, *p* = 0.008^*^^8^
Electronic cigarette use (yes/no^a^)	9 (18.8%)/39 (81.3%)	34 (15.5%)/185 (84.5%)	52 (15.4%)/286 (84.6%)	59 (11.2%)/470 (88.8%)	415 (12.6%)/2,885 (87.4%)	χ^2^(4) = 6.42, *p* = 0.170
Cannabis use (yes/no^a^)	19 (39.6%)/29 (60.4%)	47 (21.5%)/172 (78.5%)	58 (17.2%)/280 (82.8%)	55 (10.4%)/474 (89.6%)	614 (18.6%)/2,686 (81.4%)	χ^2^(4) = 38.78, *p* <0.001^*^^1, 8^
**Complacency determinants**						
Perceived susceptibility to infectious disease	3.6 (1.0)	3.5 (1.1)	3.4 (1.0)	3.7 (0.9)	3.4 (1.1)	*F*_(4, 4429)_ = 11.26, *p* <0.001^*^^4^
Perceived seriousness of COVID-19	4.1 (1.2)	4.4 (0.9)	4.5 (0.9)	4.4 (0.9)	4.5 (0.9)	*F*(_4, 3546)_ = 1.42, *p* = 0.225
Perceived safety of social distancing measures	4.0 (1.0)	3.7 (1.2)	3.5 (1.3)	3.4 (1.1)	3.6 (1.1)	*F*_(4, 3546)_ = 6.78, *p* <0.001^*^^8^
Perceived safety of going out in the community	3.1 (1.4)	3.1 (1.2)	3.0 (1.3)	2.9 (1.2)	3.1 (1.3)	*F*_(4, 3546)_ = 4.83, *p* = 0.001^*^^4^
Perceived likelihood of a second wave of COVID-19	3.9 (1.1)	4.0 (1.1)	4.2 (1.0)	4.1 (0.9)	4.0 (1.0)	*F*_(4, 3546)_ = 1.50, *p* = 0.199
Tested positive for COVID-19 (self)						χ^2^(4) = 5.34, *p* = 0.254
Tested positive	2 (4.2%)	5 (2.3%)	9 (2.7%)	7 (1.3%)	99 (3.0%)	
Not tested or tested negative^a^	46 (95.8%)	214 (97.7%)	329 (97.3%)	522 (98.7%)	3,201 (97.0%)	
Tested positive for COVID-19 (someone close)						χ^2^(4) = 40.74, *p* <0.001^*^^8^
Tested positive	14 (29.2%)	72 (32.9%)	140 (41.4%)	117 (22.1%)	923 (28.0%)	
Not tested or tested negative^a^	34 (70.8%)	147 (67.1%)	198 (58.6%)	412 (77.9%)	2,377 (72.0%)	
COVID-19 health risk factors^b^	1.0 (1.2)	0.6 (1.1)	0.6 (1.2)	0.4 (0.9)	0.8 (1.1)	*F*_(4, 4429)_ = 16.60, *p* <0.001^*^^8^
	**Mean (SD) or** ***N*** **(%)**					***t*** **(df) and** ***p*****-value**
**Confidence determinants**						
Mistrust of vaccine benefit	2.9 (1.4)	2.9 (1.4)	2.6 (1.3)	2.6 (1.1)	2.3 (1.2)	*F*_(4, 4429)_ = 18.10, *p* <0.001^*^^1, 2, 3, 4^
Worries over unforeseen future effects	4.0 (1.3)	4.1 (1.3)	3.8 (1.3)	3.8 (1.1)	3.7 (1.3)	*F*_(4, 4429)_ = 9.30, *p* <0.001^*^^2^
Concerns about commercial profiteering	3.5 (1.5)	3.7 (1.4)	3.4 (1.4)	3.2 (1.3)	2.9 (1.5)	*F*_(4, 4429)_ = 25.89, *p* <0.001^*^^2, 3, 4^
Preference for natural immunity	3.7 (1.3)	3.5 (1.3)	3.4 (1.4)	3.3 (1.2)	3.2 (1.4)	*F*_(4, 4429)_ = 6.04, *p* <0.001^*^^2^, ^3^
Positive attitudes toward holistic health approaches	12.7 (5.0)	12.8 (5.4)	12.7 (5.1)	12.7 (4.1)	11.8 (4.2)	*F*_(4, 4429)_ = 8.11, *p* <0.001^*^^2^, ^3^, ^4^
Positive attitudes toward complementary and alternative medicine	22.2 (4.3)	22.4 (4.3)	23.0 (4.2)	23.3 (3.9)	23.6 (5.0)	*F*_(4, 4429)_ = 5.77, *p* <0.001^*^^6^
Mistrust in Government's management of COVID-19	23.8 (8.7)	26.0 (9.3)	26.1 (8.6)	25.3 (8.1)	26.0 (9.1)	*F*_(4, 4429)_ = 1.45, *p* = 0.215

a *Reference variable*.

b *One point was assigned for each health risk factor (i.e., heart disease, hypertension, lung disease, diabetes, cancer, chronic kidney disease, obesity, and weakened immune system) to derive a total health risk factor score for COVID-19*.

* *p < 0.002 (0.05/29 comparisons)*.

*Bonferroni corrected p-value < 0.05 with White as the reference group:*

1 *Indigenous > Whites;*

2 *Black > Whites;*

3 *Latinx > White;*

4 *East Asian > White;*

5 *White > Indigenous;*

6 *White > Black;*

7 *White > Latinx;*

8 *White > East Asian*.

One-way analysis of variance (ANOVA) or chi-square (χ^2^) statistics were performed to examine the differences in sociodemographic, vaccine hesitancy, complacency, and confidence variables between racial groups. Participants who identified as being White were used as a reference group in all pairwise comparisons. Bonferroni correction for multiple comparisons was applied and a threshold of *p* < 0.002 (i.e., 0.05/29 comparisons) was used to establish significance. For exploratory purposes, the analyses were repeated for Canada and the United States separately. Multivariate analyses of variance (MANOVA) were subsequently performed to examine the differences in vaccine hesitancy, vaccine complacency and confidence between the racial groups, controlling for sociodemographic variables found to be significantly associated with vaccine hesitancy, including age, education, religion, region of residence, healthcare worker status, income, employment status, and political affiliation. Bonferroni correction for multiple comparisons was applied and a threshold of *p* < 0.003 (i.e., 0.05/16 comparisons) was used to establish significance. Statistical analyses were performed using IBM SPSS Statistics (version 26, IBM Corp., Armonk, N.Y., USA). The EQUATOR Reporting Guidelines were followed. Additional survey details can be found in Supplementary Material 1 and the full list of variables and data collected for the survey are available online at http://www.covid19-database.com.

## Results

**Figure 1:**
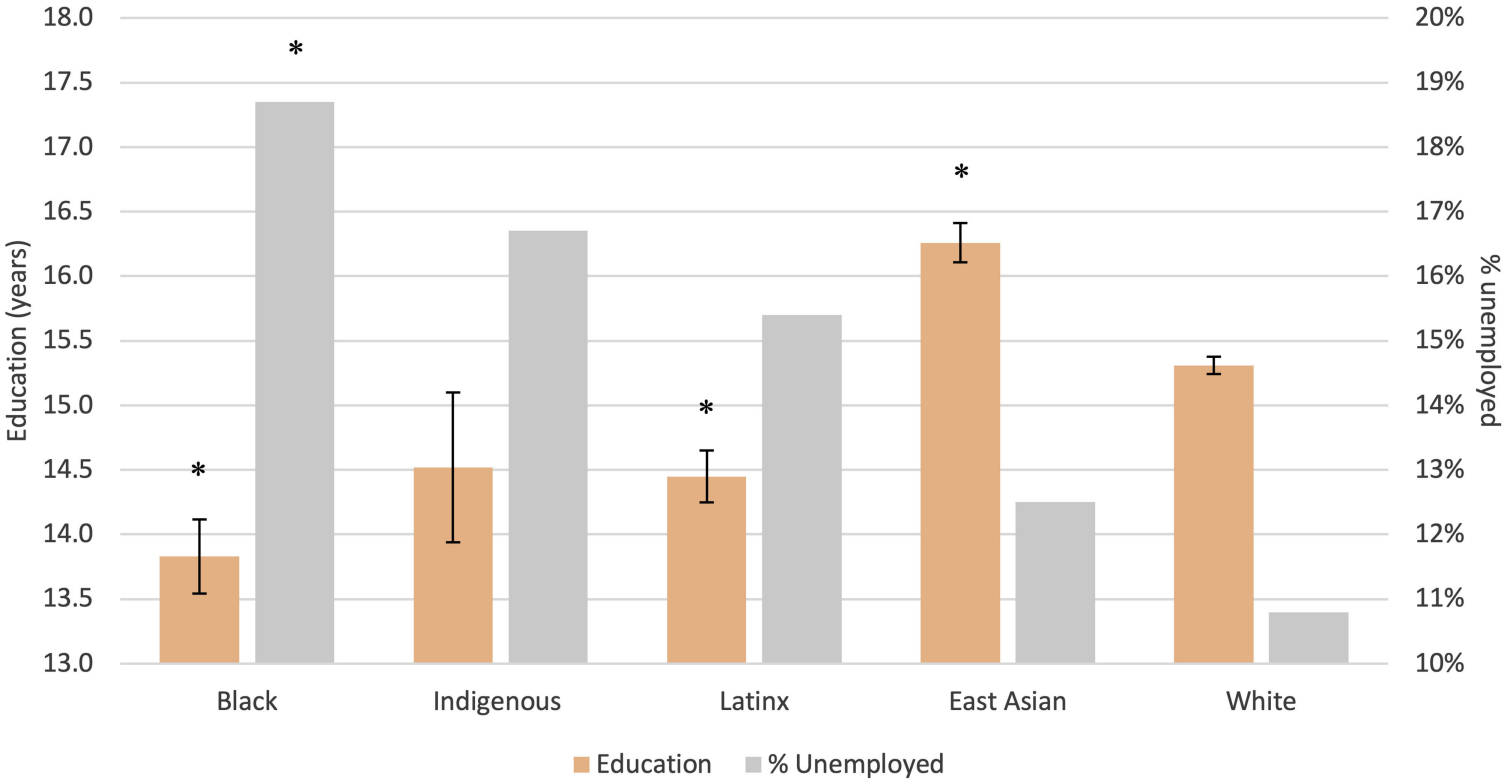
Differences in years of education and % unemployed between racial groups. *Bonferroni corrected *p*-value < 0.05 with White as the reference group; Error bars represent standard error.

The mean age was 48.7 (SD = 17.2) and 50.4% of the participants were women. The majority of participants were White (74.4%). One percent of the participants were Indigenous, 4.9% Black, 11.9% East Asian, and 7.6% Latinx. Indigenous, Black, and Latinx participants were more socioeconomically disadvantaged than East Asian and White participants. Sociodemographic characteristics of the participants included in the study can be found in Table 1; Figure 1; Supplementary Materials 2, 3.

**Figure 2:**
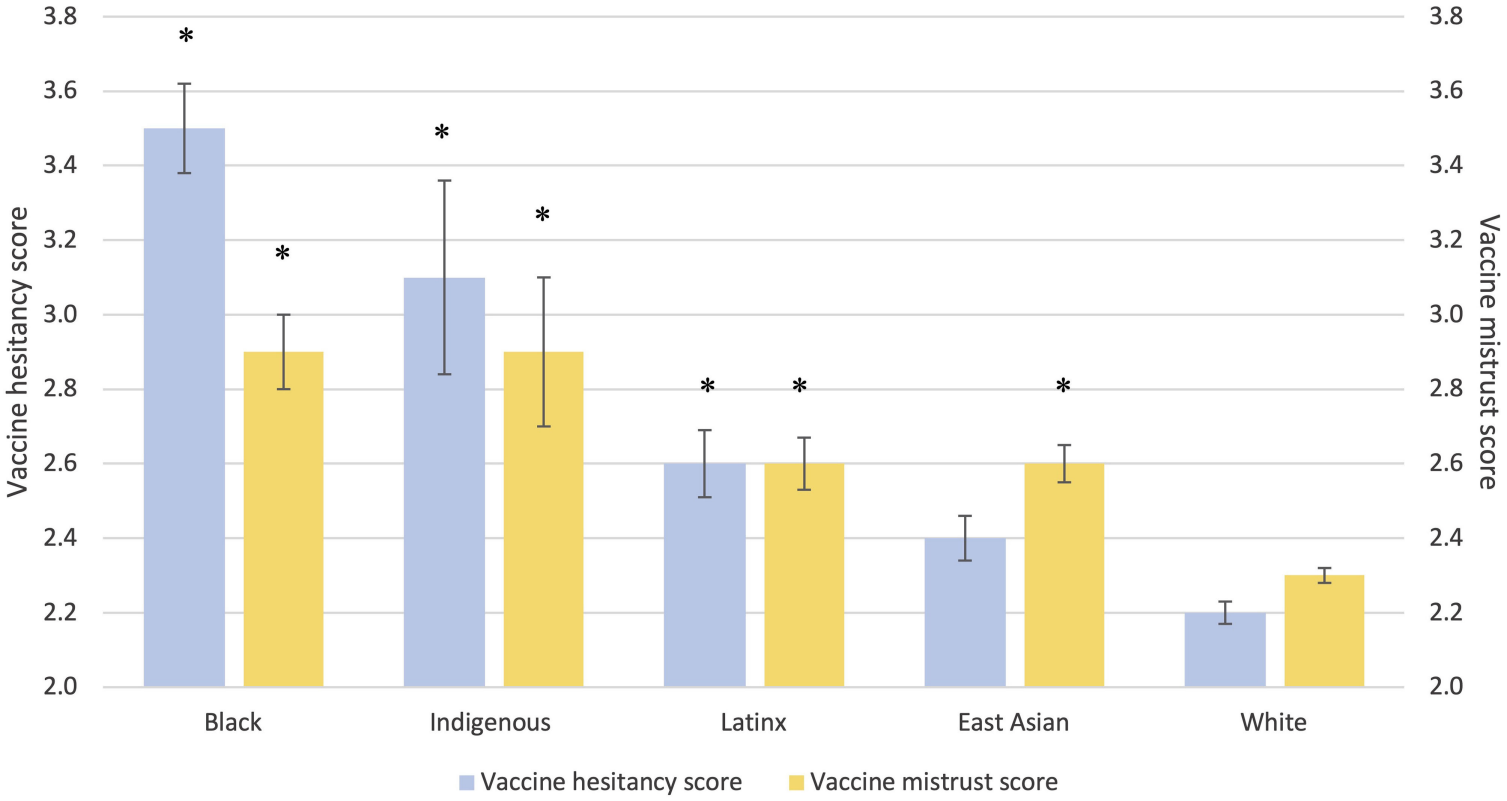
Differences in vaccine hesitancy and vaccine mistrust scores between racial groups. *Bonferroni corrected *p*-value < 0.05 with White as the reference group; Error bars represent standard error.

In the unadjusted analyses, vaccine hesitancy was significantly higher in Black, Indigenous, and Latinx compared to White participants (Table 1; Figure 2). When controlling for sociodemographic factors, the group difference in vaccine hesitancy remained for Indigenous and Black vs. White participants, but not between Latinx and White participants. Separate unadjusted analyses by country showed higher vaccine hesitancy in Black compared to White participants in both Canada and the U.S., but no significant differences between Latinx and White participants in Canada and between Indigenous and White participants in the U.S. (Supplementary Materials 4–7).

In terms of determinants of vaccine complacency, disproportionately affected racial minority groups perceived COVID-19 with the same degree of seriousness as White participants. East Asian participants were more likely than White participants to believe they are susceptible to infectious disease and less likely to perceive the current social distancing and community restrictions to be safe and restrictive enough. East Asian participants also had a fewer number of health risk factors for COVID-19 compared to White participants. The group differences in the determinants of vaccine complacency remained when controlling for sociodemographic differences, with the exception of health risk factors for COVID-19, which became non-significant.

Vaccine confidence was generally lower in all racial minority groups compared to White participants. Attitudes toward vaccinations, including mistrust in vaccine benefit (Figure 2), worries over unforeseen future effects of vaccines, concerns about commercial profiteering, and preference for natural immunity were generally higher in disproportionately affected racial minorities compared to White participants. Black, Latinx, and East Asian participants had more positive attitudes toward holistic health approaches compared to White participants, although attitudes toward complementary and alternative medicine were more positive in White compared to Black participants. There were no group differences with respect to trust in Government's management of COVID-19. Group differences in the determinants of vaccine confidence remained when controlling for sociodemographic differences (Supplementary Material 8).

## Discussion

Addressing vaccine hesitancy prior to the availability of vaccines for COVID-19 is essential to achieve equitable herd immunity among racial minorities who have been disproportionately affected by COVID-19. At the time of this study, only 43.7% of Indigenous, 33.4% of Black, and 56.5% of Latinx are “very probably” to “definitely” likely to get a COVID-19 vaccine, as compared to 59.6% of East Asians and 67.4% of Whites.

Racial minorities had lower vaccine confidence, while no notable group differences were found in vaccine complacency. In other words, all groups viewed COVID-19 with the same degree of seriousness, yet differed in their degree of vaccine confidence. In the current sample, racial minority groups disproportionately affected by COVID-19 were more socioeconomically disadvantaged and more likely to be personally affected by COVID-19. Disproportionately affected groups had lower years of education, higher unemployment, and less income, and were also generally younger, more religious, and less conservative than White participants. Notably, group differences in vaccine hesitancy between Indigenous and Black compared to White participants remained after accounting for these sociodemographic differences. The persistence of group differences after accounting for socioeconomic disparities may reflect the historical and contemporary systemic factors that contribute to mistrust in medical interventions among racial minorities in North America. These include the Tuskegee Experiment where Black American men were deceived subjects of an observation study of untreated syphilis and the Qu'Appelle BCG Vaccine Trial in which First Nations children of the Qu'Appelle reserves in southern Saskatchewan were subjects of a vaccine trial for tuberculosis, while their impoverished living conditions were left unaddressed (17, 18).

There are a few limitations to this study. First, only English-speaking participants who are familiar with using a computer were included. Second, the sample size for Black, Indigenous, and Latinx participants was relatively low compared to East Asian and White participants. As such, some of the null results may be attributed to a lack of statistical power. Third, we recognize that the use of racial and ethnic categorizations as employed in this study are imperfect. Participants had the opportunity to select the category they most identified with, which was felt to be the best means to overcome this limitation where only a single response option was available. Participants were also offered the option of choosing “other” if they did not feel one of the categories represented them. It is possible some participants may not identify with the nomenclature of the racial and ethnic categories and thus were not included in the study. Fourth, the study included a convenience sample and thus may not be representative of the general population. Lastly, the efficacy and the specific risks associated with the COVID-19 vaccines were unknown at the time of the study.

In summary, disproportionately affected racial minority groups may have higher vaccine hesitancy, in particular lower COVID-19 vaccine confidence. If the societal objective is to ensure the equitable attainment of herd immunity among racial minority communities disproportionately affected by COVID-19, in addition to optimizing vaccine accessibility [i.e., ensuring vaccines are easily accessible and affordable (14)], special efforts ought to be made within these communities to bolster vaccine confidence using a culturally sensitive, community centered approach. Moreover, “in times of famine and pestilence,” local and national governments may have the legal responsibility to achieve this aim (19).

## Data Availability Statement

The raw data supporting the conclusions of this article are available, without undue reservation, at http://www.covid19-database.com.

## Ethics Statement

All studies involving human participants are reviewed and approved by the Centre for Addiction and Mental Health. Participants provided their written informed consent to participate in this study.

## Author Contributions

PG formulated the research aims, designed the research methodology, provided oversight in executing the study, and wrote the first draft of the manuscript. JK conducted statistical analyses and assisted with writing the manuscript. LQ assisted with designing the study and interpreting the data. SW assisted with designing the study and edited the manuscript. EB assisted with formulating the research aims, designing research methodology, validating research outputs, and editing the manuscript. BA assisted with formulating the research aims, interpreting the data, and edited the manuscript. BP assisted with interpreting the data and edited the manuscript. AG-G provided oversight in executing the study and edited the manuscript. All authors contributed to the article and approved the submitted version.

## Funding

This work was supported by the CAMH Foundation, an Academic Scholars Award from the Department of Psychiatry, University of Toronto, and Canadian Institute of Health Research (CIHR) (PJT-159807 to PG).

## Conflict of Interest

PG reports receiving research support from the Canadian Institute of Health Research (CIHR), Ontario Ministry of Health and Long-Term Care, Ontario Mental Health Foundation (OMHF), and the Centre for Addiction and Mental Health (CAMH). AG-G has received support from the United States National Institute of Health, CIHR, OMHF, Consejo Nacional de Ciencia y Tecnologia, the Instituto de Ciencia y Tecnologia del DF, the Brain & Behavior Research Foundation (Formerly NARSAD), the Ontario Ministry of Health and Long-Term Care, the Ontario Ministry of Research and Innovation Early Research Award, and Janssen. The remaining authors declare that the research was conducted in the absence of any commercial or financial relationships that could be construed as a potential conflict of interest.

## Publisher's Note

All claims expressed in this article are solely those of the authors and do not necessarily represent those of their affiliated organizations, or those of the publisher, the editors and the reviewers. Any product that may be evaluated in this article, or claim that may be made by its manufacturer, is not guaranteed or endorsed by the publisher.
