# Supplementary material for: Vaccine Hesitancy Is a Barrier to Achieving Equitable Herd Immunity Among Racial Minorities
Source: Front Med (Lausanne). 2021 Nov 24;8:668299. doi: 10.3389/fmed.2021.668299 (PMC8652048; doi:10.3389/fmed.2021.668299)
Supplement: Supplementary file 1 [file Data_Sheet_1.PDF]

Supplemental Material 1. EQUATOR Reporting Guidelines: Checklist for Reporting Results of Internet E-Surveys (CHERRIES)

Design and Institutional Review Board approval

We collected data from participants aged 18 years or older from the most populous U.S. states including New York, California, Florida, and Texas and from English-speaking Canada between May to August 2020. We utilized an open-survey design. The study was approved by our institution's Research Ethics Board (REB). All participants provided informed consent prior to starting the survey. No identifiable information was collected.

Development and pre-testing

The survey was developed, pre-tested, and collected using a web-based platform *Dynata*, a global market research company. The survey was also tested by members of our team to ensure usability and technical functionality prior to the soft launch. Soft launch data was reviewed prior to the hard launch by our team and by Dynata.

Recruitment process and survey administration

Participation in this study was voluntary and open to all participants in Canada and the United states. Participants were recruited via email based on demographic profile. Participants were also able to take the survey by accessing Dynata's survey platform. Participants received panel credit points, which they can redeem as coupons, for their time and participation. Responses were collected between May to August 2020.

Some of the survey items were randomized to prevent response bias. In order to reduce the number of questions asked, certain questions were only asked conditionally. A maximum of one survey questionnaire was asked per screen. The number of items per questionnaire ranged from 1 to a maximum of 24 questions. The survey questions were distributed across 97 screens. We enforced mandatory single response selection throughout the survey to ensure that participant does not select multiple answer options. Completeness checks were done per screen to prevent participants from skipping questions. Participants were not allowed to go back to previous screens to change their answers once their answers were submitted.

Quality Control, Response Rate, and Analysis

In order to prevent the same respondents from completing the survey multiple times, IP address and digital fingerprint were tracked, and de-deduplication checks were performed. Manual open end verbatim checks were put in place to identify and remove any poor-quality data. A racer check was put in place to remove those who completed the survey in less than 30% of the median survey length. Only completed questionnaires were considered for analysis.

Survey attempts not included in the study were removed for the following reasons: over quota (n=44), partial or complete dropouts (n=1686), not meeting our age and region quota (n=514), and not passing open end verbatim checks (n=362) and completing the survey too quickly (n=104).

Supplemental Material 2. Differences in years of education and unemployment between racial groups in Canada.

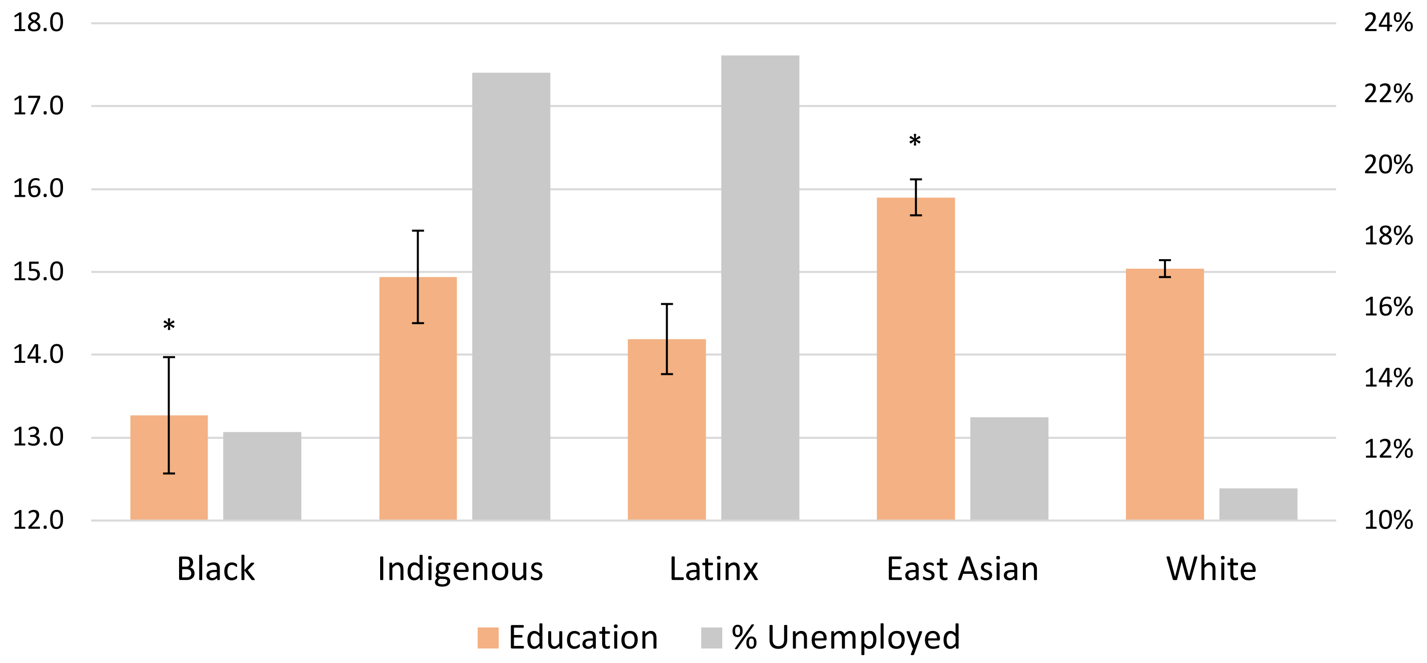

*Bonferroni corrected p-value with White as the reference group; Error bars represent standard error

Supplemental Material 3. Differences in years of education and unemployment between racial groups in the United States.

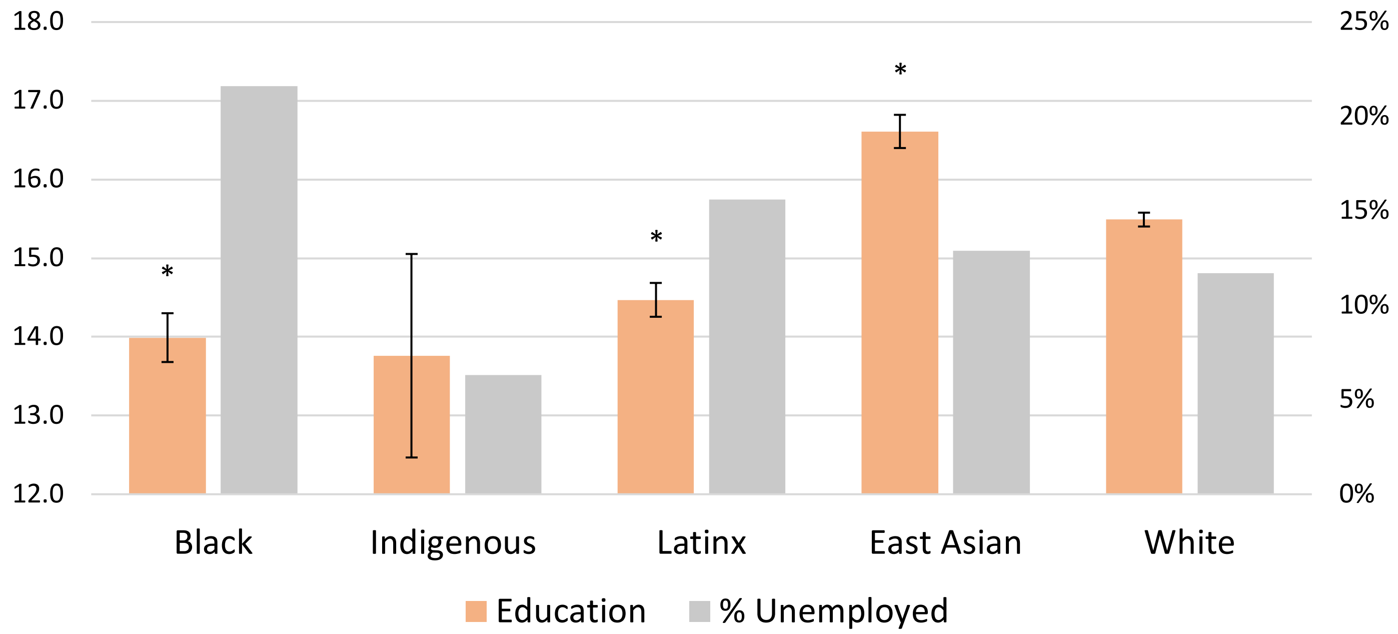

*Bonferroni corrected p-value with White as the reference group; Error bars represent standard error

Supplemental Material 4. Differences in sociodemographic and vaccine hesitancy, complacency, and confidence determinants between racial groups in Canada.

	Indigenous (N=31)	Black (N=48)	Latinx (N=26)	East Asian (N=259)	White (N=1316)	
			Mean (SD) or N (%)			t(df) and p-value
Vaccine hesitancy score	3.1 (1.8)	3.4 (1.7)	2.6 (1.7)	2.5 (1.5)	2.2 (1.5)	F(4,1679)=11.63, p<0.001*[1,2]
**Sociodemographic determinants**						
Age	39.0 (14.0)	36.7 (16.2)	32.7 (16.3)	38.1 (13.9)	50.2 (16.7)	F(4,1679)=43.68, p<0.001*[5,6,7,8]
Gender (man/woman[a])	15 (50.0%) /15 (50.0%)	26 (54.2%) /22 (45.8%)	11 (44.0%) /14 (56.0%)	130 (50.4%) /128 (49.6%)	651 (49.7%) /659 (50.3%)	$\chi^2(4)$=0.74, p=0.946
Education (years)	14.9 (3.1)	13.3 (4.9)	14.2 (2.2)	15.9 (3.5)	15.0 (3.7)	F(4,1678)=6.57, p<0.001*[4,6]
Religion (yes/no[a])	11 (40.7%) /16 (59.3%)	36 (80.0%) /9 (20.0%)	18 (69.2%) /8 (30.8%)	95 (38.5%) /152 (61.5%)	750 (58.9%) /523 (41.1%)	$\chi^2(4)$=50.17, p<0.001*[8]
Population density						
1,000 or less[a]	3 (10.7%)	1 (2.6%)	0 (0.0%)	3 (1.3%)	68 (5.7%)	-
1,000 to 29,999	5 (17.9%)	6 (15.4%)	1 (4.0%)	6 (2.6%)	190 (16.0%)	$\chi^2(4)$=1.61, p=0.807
30,000 to 99,999	9 (32.1%)	9 (23.1%)	5 (20.0%)	25 (10.7%)	204 (17.2%)	$\chi^2(4)$=5.49, p=0.241
100,000 or more	11 (39.3%)	23 (59.0%)	19 (76.0%)	199 (85.4%)	726 (61.1%)	$\chi^2(4)$=18.30, p=0.001*[4]
Household income						
less than $20,000[a]	1 (3.4%)	5 (11.4%)	0 (0.0%)	10 (4.2%)	72 (5.8%)	-
$20,000 - $59,999	11 (37.9%)	15 (34.1%)	11 (50.0%)	55 (22.9%)	373 (30.2%)	$\chi^2(4)$=3.87, p=0.424
$60,000 - $99,999	7 (24.1%)	11 (25.0%)	8 (36.4%)	87 (36.3%)	398 (32.2%)	$\chi^2(4)$=6.54, p=162
$100,000 - $139,999	7 (24.1%)	11 (25.0%)	2 (9.1%)	49 (20.4%)	234 (18.9%)	$\chi^2(4)$=2.97, p=0.563
$140,000 or more	3 (10.3%)	2 (4.5%)	1 (4.5%)	39 (16.3%)	160 (12.9%)	$\chi^2(4)$=8.46, p=0.076
Employment status						
Unemployed	7 (22.6%)	6 (12.5%)	6 (23.1%)	32 (12.9%)	135 (10.9%)	$\chi^2(4)$=6.00, p=0.200
Employed[a]	22 (71.0%)	28 (58.3%)	10 (38.5%)	171 (69.0%)	667 (54.0%)	-
Student	0 (0.0%)	11 (22.9%)	7 (26.9%)	25 (10.1%)	43 (3.5%)	$\chi^2(4)$=53.43, p<0.001*[2,3,4]
Retired	2 (6.5%)	3 (6.3%)	3 (11.5%)	20 (8.1%)	391 (31.6%)	$\chi^2(4)$=66.57, p<0.001*[8]
Healthcare worker (yes/no[a])	3 (9.7%) /28 (90.3%)	11 (22.9%) /37 (77.1%)	6 (23.1%) /20 (76.9%)	47 (18.1%) /212 (81.9%)	134 (10.2%) /1182 (89.8%)	$\chi^2(4)$=22.04, p<0.001*[8]
Political spectrum						
Communism left wing or socialism	1 (3.2%)	3 (6.3%)	2 (7.7%)	7 (2.7%)	103 (7.8%)	$\chi^2(4)$=10.22, p=0.037
Liberal	9 (29.0%)	12 (25.0%)	13 (50.0%)	83 (32.0%)	358 (27.2%)	$\chi^2(4)$=7.24, p=0.124
Center[a]	13 (41.9%)	21 (43.8%)	5 (19.2%)	99 (38.2%)	477 (36.2%)	-
Conservative	6 (19.4%)	8 (16.7%)	6 (23.1%)	65 (25.1%)	367 (27.9%)	$\chi^2(4)$=5.10, p=0.277
Fascism right wing or authoritarianism	2 (6.5%)	4 (8.3%)	0 (0.0%)	5 (1.9%)	11 (0.8%)	$\chi^2(4)$=19.05, p<0.001*[2]
Alcohol use (yes/no[a])	15 (48.4%) /16 (51.6%)	28 (58.3%) /20 (41.7%)	17 (65.4%) /9 (34.6%)	150 (57.9%) /109 (42.1%)	923 (70.1%) /393 (29.9%)	$\chi^2(4)$=22.05, p<0.001*[5,8]

Cigarette use (yes/no[a])	7 (22.6%) /24 (77.4%)	12 (25.0%) /36 (75.0%)	4 (15.4%) /22 (84.6%)	43 (16.6%) /216 (83.4%)	283 (21.5%) /1033 (78.5%)	$\chi^2(4)=4.21$, p=0.379
Electronic cigarette use (yes/no[a])	9 (29.0%) /22 (71.0%)	13 (27.1%) /35 (72.9%)	5 (19.2%) /21 (80.8%)	31 (12.0%) /228 (88.0%)	152 (11.6%) /1164 (88.4%)	$\chi^2(4)=19.31$, p=0.001*
Cannabis use (yes/no[a])	14 (45.2%) /17 (54.8%)	17 (35.4%) /31 (64.6%)	9 (34.6%) /17 (65.4%)	37 (14.3%) /222 (85.7%)	310 (23.6%) /1006 (76.4%)	$\chi^2(4)=26.06$, p<0.001*[1]
**Complacency determinants**						
Perceived susceptibility to infectious disease	3.9 (0.7)	3.7 (1.0)	3.3 (1.0)	3.7 (0.9)	3.4 (1.1)	F(4,1679)=6.39, p<0.001*[4]
Perceived seriousness of COVID-19	4.3 (1.0)	4.3 (0.8)	4.0 (0.7)	4.3 (0.9)	4.5 (0.9)	F(4,796)=1.86, p=0.116
Perceived safety of social distancing measures	4.2 (0.8)	3.4 (1.2)	3.2 (1.2)	3.5 (1.0)	3.8 (1.0)	F(4,796)=3.92, p=0.004
Perceived safety of going out in the community	3.5 (1.1)	3.3 (1.3)	3.2 (1.0)	3.1 (1.1)	3.3 (1.1)	F(4,796)=1.47, p=0.209
Perceived likelihood of a second wave of COVID-19	4.1 (0.7)	3.8 (1.3)	3.9 (0.9)	3.9 (0.9)	4.1 (0.9)	F(4,796)=1.13, p=0.341
Tested positive for COVID-19 (self)						$\chi^2(4)=3.14$, p=0.535
Tested positive	2 (6.5%)	2 (4.2%)	0 (0.0%)	6 (2.3%)	33 (2.5%)	
Not tested or tested negative[a]	29 (93.5%)	46 (95.8%)	26 (100.0%)	253 (97.7%)	1283 (97.5%)	
Tested positive for COVID-19 (someone close)						$\chi^2(4)=14.23$, p=0.007
Tested positive	7 (22.6%)	12 (25.0%)	10 (38.5%)	41 (15.8%)	200 (15.2%)	
Not tested or tested negative[a]	24 (77.4%)	36 (75.0%)	16 (61.5%)	218 (84.2%)	1116 (84.8%)	
COVID-19 health risk factors[b]	0.9 (1.0)	0.5 (1.2)	0.3 (0.6)	0.4 (1.0)	0.8 (1.1)	F(4,1679)=7.44, p<0.001*[8]
**Confidence determinants**						
Mistrust of vaccine benefit	3.0 (1.3)	3.1 (1.5)	2.9 (1.5)	2.6 (1.0)	2.3 (1.2)	F(4,1679)=9.38, p<0.001*[1,2,4]
Worries over unforeseen future effects	3.9 (1.4)	4.0 (1.5)	3.6 (1.1)	3.8 (1.1)	3.6 (1.3)	F(4,1679)=4.40, p=0.002
Concerns about commercial profiteering	3.5 (1.4)	3.6 (1.4)	3.2 (1.2)	3.2 (1.3)	2.8 (1.5)	F(4,1679)=11.08, p<0.001*[1,2,4]
Preference for natural immunity	3.6 (1.4)	3.5 (1.5)	3.1 (1.2)	3.3 (1.2)	3.1 (1.4)	F(4,1679)=3.02, p=0.017
Positive attitudes toward holistic health approaches	13.7 (5.0)	13.1 (5.6)	12.8 (4.8)	12.4 (4.2)	11.8 (4.2)	F(4,1679)=3.93, p=0.004
Positive attitudes toward complementary and alternative medicine	21.9 (4.9)	23.3 (4.0)	22.7 (4.6)	23.7 (3.9)	24.3 (5.2)	F(4,1679)=3.37, p=0.009
Mistrust in Government's management of COVID-19	21.6 (6.7)	20.8 (7.6)	20.7 (5.0)	21.2 (6.2)	21.4 (6.9)	F(4,1679)=0.22, p=0.927

[a]Reference variable.

[b]One point was assigned for each health risk factor (i.e., heart disease, hypertension, lung disease, diabetes, cancer, chronic kidney disease, obesity, and weakened immune system) to derive a total health risk factor score for COVID-19.

*p<0.002 (0.05/29 comparisons)

Bonferroni corrected p-value <0.05 with Whites as the reference group:

[1]Indigenous > Whites; [2]Black > Whites; [3]Latinx > White; [4]East Asian > White; [5]White > Indigenous; [6]White > Black; [7]White > Latinx; [8]White > East Asian.

Supplemental Material 5. Differences in sociodemographic and vaccine hesitancy, complacency, and confidence determinants between racial groups in the United States.

	Indigenous (N=17)	Black (N=171)	Latinx (N=312)	East Asian (N=270)	White (N=1984)	
			Mean (SD) or N (%)			t(df) and p-value
Vaccine hesitancy score	3.1 (1.9)	3.5 (1.8)	2.6 (1.7)	2.3 (1.5)	2.2 (1.5)	F(4,2753)=29.59, p<0.001*[2,3]
**Sociodemographic determinants**						
Age	51.5 (20.1)	41.3 (17.5)	41.0 (17.0)	41.5 (14.3)	52.5 (16.7)	F(4,2753)=63.12, p<0.001*[6,7,8]
Gender (man/woman[a])	9 (52.9%) /8 (47.1%)	64 (37.6%) /106 (62.4%)	136 (43.6%) /176 (56.4%)	110 (40.9%) /159 (59.1%)	1030 (52.1%) /947 (47.9%)	$\chi^2(4)$=27.18, p<0.001*[6,8]
Education (years)	13.8 (5.3)	14.0 (4.1)	14.5 (3.8)	16.6 (3.5)	15.5 (3.9)	F(4,2753)=17.66, p<0.001*[4,6,7]
Religion (yes/no[a])	11 (73.3%) /4 (26.7%)	124 (75.2%) /41 (24.8%)	232 (78.6%) /63 (21.4%)	120 (46.7%) /137 (53.3%)	1383 (72.2%) /532 (27.8%)	$\chi^2(4)$=84.16, p<0.001*[3,8]
Population density						
1,000 or less[a]	1 (5.9%)	5 (3.3%)	6 (2.2%)	2 (0.8%)	35 (2.0%)	-
1,000 to 29,999	2 (11.8%)	10 (6.6%)	26 (9.7%)	14 (5.9%)	206 (11.5%)	$\chi^2(4)$=4.75, p=0.314
30,000 to 99,999	4 (23.5%)	27 (17.8%)	49 (18.2%)	43 (18.1%)	311 (17.4%)	$\chi^2(4)$=3.23, p=0.521
100,000 or more	10 (58.8%)	110 (72.4%)	188 (69.9%)	179 (75.2%)	1235 (69.1%)	$\chi^2(4)$=4.64, p=0.326
Household income						
less than $20,000[a]	2 (11.8%)	34 (20.7%)	48 (16.1%)	14 (5.5%)	106 (5.6%)	-
$20,000 - $59,999	8 (47.1%)	62 (37.8%)	105 (35.1%)	61 (23.8%)	397 (21.1%)	$\chi^2(4)$=14.82, p=0.005
$60,000 - $99,999	5 (29.4%)	42 (25.6%)	82 (27.4%)	72 (28.1%)	498 (26.5%)	$\chi^2(4)$=47.46, p<0.001*[6,7]
$100,000 - $139,999	2 (11.8%)	9 (5.5%)	32 (10.7%)	45 (17.6%)	363 (19.3%)	$\chi^2(4)$=95.25, p<0.001*[6,7]
$140,000 or more	0 (0.0%)	17 (10.4%)	32 (10.7%)	64 (25.0%)	515 (27.4%)	$\chi^2(4)$=129.97, p<0.001*[6,7]
Employment status						
Unemployed	1 (6.3%)	35 (21.6%)	46 (15.6%)	34 (12.9%)	223 (11.7%)	$\chi^2(4)$=14.08, p=0.007
Employed[a]	8 (50.0%)	84 (51.9%)	170 (57.8%)	181 (68.6%)	1095 (57.7%)	-
Student	1 (6.3%)	16 (9.9%)	39 (13.3%)	26 (9.8%)	51 (2.7%)	$\chi^2(4)$=66.86, p<0.001*[2,3,4]
Retired	6 (37.5%)	27 (16.7%)	39 (13.3%)	23 (8.7%)	530 (27.9%)	$\chi^2(4)$=54.89, p<0.001*[7,8]
Healthcare worker (yes/no[a])	2 (11.8%) /15 (88.2%)	39 (22.8%) /132 (77.2%)	50 (16.0%) /262 (84.0%)	44 (16.3%) /226 (83.7%)	245 (12.3%) /1739 (87.7%)	$\chi^2(4)$=17.95, p=0.001*[2,6]
Political spectrum						
Communism left wing or socialism	0 (0.0%)	7 (4.1%)	18 (5.8%)	11 (4.1%)	100 (5.0%)	$\chi^2(4)$=5.50, p=0.240
Liberal	5 (29.4%)	61 (35.7%)	90 (28.8%)	82 (30.4%)	587 (29.6%)	$\chi^2(4)$=12.45, p=0.014
Center[a]	3 (17.6%)	71 (41.5%)	137 (43.9%)	118 (43.7%)	590 (29.7%)	-
Conservative	9 (52.9%)	25 (14.6%)	64 (20.5%)	55 (20.4%)	658 (33.2%)	$\chi^2(4)$=71.74, p<0.001*[6,7,8]
Fascism right wing or authoritarianism	0 (0.0%)	7 (4.1%)	3 (1.0%)	4 (1.5%)	49 (2.5%)	$\chi^2(4)$=8.98, p=0.062
Alcohol use (yes/no[a])	8 (47.1%) /9 (52.9%)	91 (53.2%) /80 (46.8%)	172 (55.1%) /140 (44.9%)	127 (47.0%) /143 (53.0%)	1344 (67.7%) /640 (32.3%)	$\chi^2(4)$=65.94, p<0.001*[5,6,7,8]

Cigarette use (yes/no[a])	3 (17.6%) /14 (82.4%)	28 (16.4%) /143 (83.6%)	54 (17.3%) /258 (82.7%)	29 (10.7%) /241 (89.3%)	383 (19.3%) /1601 (80.7%)	χ^2(4)=12.31, p=0.015
Electronic cigarette use (yes/no[a])	0 (0.0%) /17 (100.0%)	21 (12.3%) /150 (87.7%)	47 (15.1%) /265 (84.9%)	28 (10.4%) /242 (89.6%)	263 (13.3%) /1721 (86.7%)	χ^2(4)=5.54, p=0.236
Cannabis use (yes/no[a])	5 (29.4%) /12 (70.6%)	30 (17.5%) /141 (82.5%)	49 (15.7%) /263 (84.3%)	18 (6.7%) /252 (93.3%)	304 (15.3%) /1680 (84.7%)	χ^2(4)=18.75, p<0.001*[1,8]
**Complacency determinants**						
Perceived susceptibility to infectious disease	3.1 (1.2)	3.5 (1.1)	3.5 (1.0)	3.7 (1.0)	3.4 (1.1)	F(4,2753)=6.63, p<0.001*[4]
Perceived seriousness of COVID-19	4.0 (1.3)	4.4 (0.9)	4.5 (0.9)	4.5 (0.9)	4.4 (0.9)	F(4,2753)=1.33, p=0.255
Perceived safety of social distancing measures	3.9 (1.3)	3.7 (1.3)	3.5 (1.3)	3.3 (1.1)	3.6 (1.2)	F(4,2753)=5.15, p<0.001*[8]
Perceived safety of going out in the community	2.8 (1.6)	3.1 (1.2)	2.9 (1.3)	2.7 (1.2)	3.1 (1.3)	F(4,2753)=4.06, p=0.003
Perceived likelihood of a second wave of COVID-19	3.7 (1.4)	4.0 (1.1)	4.2 (1.0)	4.2 (0.9)	4.1 (1.0)	F(4,2753)=2.54, p=0.038
Tested positive for COVID-19 (self)						χ^2(4)=8.81, p=0.066
Tested positive	0 (0.0%)	3 (1.8%)	9 (2.9%)	1 (0.4%)	66 (3.3%)	
Not tested or tested negative[a]	17 (100.0%)	168 (98.2%)	303 (97.1%)	269 (99.6%)	1918 (96.7%)	
Tested positive for COVID-19 (someone close)						χ^2(4)=11.94, p=0.018
Tested positive	7 (41.2%)	60 (35.1%)	130 (41.7%)	76 (28.1%)	723 (36.4%)	
Not tested or tested negative[a]	10 (58.8%)	111 (64.9%)	182 (58.3%)	194 (71.9%)	1261 (63.6%)	
COVID-19 health risk factors[b]	1.4 (1.4)	0.6 (1.1)	0.7 (1.2)	0.4 (0.8)	0.8 (1.1)	F(4,2753)=10.37, p<0.001*[8]
**Confidence determinants**						
Mistrust of vaccine benefit	2.6 (1.5)	2.9 (1.4)	2.5 (1.3)	2.5 (1.1)	2.3 (1.2)	F(4,2753)=9.75, p<0.001*[2]
Worries over unforeseen future effects	4.1 (1.2)	4.1 (1.2)	3.8 (1.3)	3.8 (1.2)	3.7 (1.2)	F(4,2753)=5.11, p<0.001*[2]
Concerns about commercial profiteering	3.4 (1.6)	3.7 (1.3)	3.4 (1.4)	3.1 (1.3)	2.9 (1.5)	F(4,2753)=14.46, p<0.001*[2,3]
Preference for natural immunity	3.8 (1.4)	3.5 (1.3)	3.5 (1.4)	3.2 (1.3)	3.2 (1.4)	F(4,2753)=3.73, p=0.005
Positive attitudes toward holistic health approaches	10.8 (4.4)	12.7 (5.3)	12.7 (5.1)	12.9 (4.0)	11.9 (4.2)	F(4,2753)=5.75, p<0.001*[3,4]
Positive attitudes toward complementary and alternative medicine	22.8 (3.1)	22.1 (4.3)	23.0 (4.2)	22.9 (4.0)	23.2 (4.7)	F(4,2753)=2.36, p=0.051
Mistrust in Government's management of COVID-19	27.8 (10.5)	27.5 (9.2)	26.5 (8.6)	29.2 (7.7)	29.0 (9.1)	F(4,2753)=6.18, p<0.001*[7]

[a]Reference variable.

[b]One point was assigned for each health risk factor (i.e., heart disease, hypertension, lung disease, diabetes, cancer, chronic kidney disease, obesity, and weakened immune system) to derive a total health risk factor score for COVID-19.

*p<0.002 (0.05/29 comparisons)

Bonferroni corrected p-value <0.05 with Whites as the reference group:

[1]Indigenous > Whites; [2]Black > Whites; [3]Latinx > White; [4]East Asian > White; [5]White > Indigenous; [6]White > Black; [7]White > Latinx; [8]White > East Asian.

Supplemental Material 6. Differences in vaccine hesitancy and vaccine mistrust scores between racial groups in Canada.

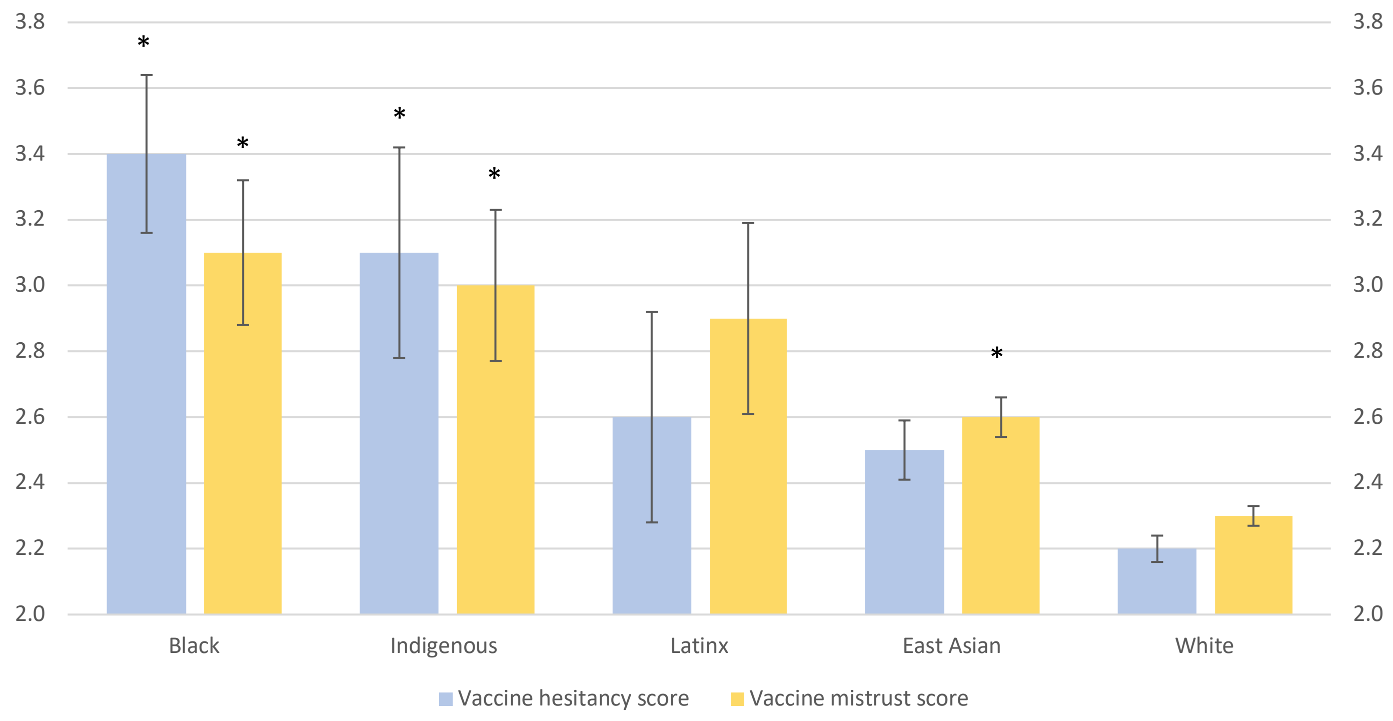

*Bonferroni corrected p-value with White as the reference group; Error bars represent standard error

Supplemental Material 7. Differences in vaccine hesitancy and vaccine mistrust scores between racial groups in the United States.

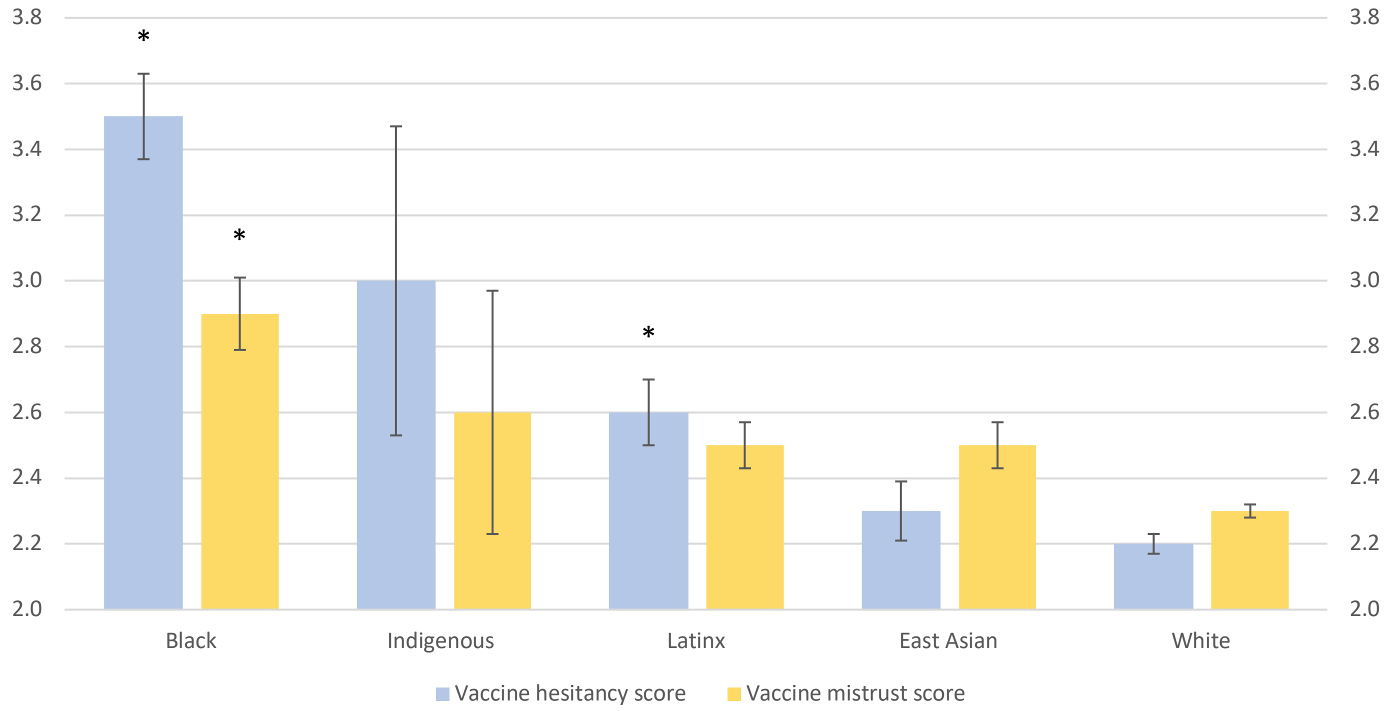

*Bonferroni corrected p-value with White as the reference group; Error bars represent standard error

Supplemental Material 8. Differences in vaccine hesitancy, complacency, and confidence determinants between racial groups, controlling for sociodemographic differences, including age, education, religion (yes/no), region of residence, healthcare worker (yes/no), income, employment status, and political affiliation.

	Indigenous	Black	Latinx	East Asian	White	
Vaccine hesitancy score	3.1 (1.8)	3.5 (1.8)	2.6 (1.7)	2.4 (1.5)	2.2 (1.5)	F(4,4025)=26.87, p<0.001*[1,2]
**Complacency determinants**			**Mean (SD) or N (%)**			**t(df) and p-value**
Perceived susceptibility to infectious disease	3.6 (1.0)	3.5 (1.1)	3.4 (1.0)	3.7 (0.9)	3.4 (1.1)	F(4,3226)=3.99, p=0.003
Perceived seriousness of COVID-19	4.1 (1.2)	4.4 (0.9)	4.5 (0.9)	4.4 (0.9)	4.5 (0.9)	F(4,3226)=1.38, p=0.239
Perceived safety of social distancing measures	4.0 (1.0)	3.7 (1.2)	3.5 (1.3)	3.4 (1.1)	3.6 (1.1)	F(4,3226)=2.53, p=0.038
Perceived safety of going out in the community	3.1 (1.4)	3.1 (1.2)	3.0 (1.3)	2.9 (1.2)	3.1 (1.3)	F(4,3226)=6.56, p<0.001*[4]
Perceived likelihood of a second wave of COVID-19	3.9 (1.1)	4.0 (1.1)	4.2 (1.0)	4.1 (0.9)	4.0 (1.0)	F(4,3226)=1.20, p=0.309
Tested positive for COVID-19 (self)						F(4,3226)=4.01, p=0.003
Tested positive	2 (4.2%)	5 (2.3%)	9 (2.7%)	7 (1.3%)	99 (3.0%)	
Not tested or tested negative[a]	46 (95.8%)	214 (97.7%)	329 (97.3%)	522 (98.7%)	3201 (97.0%)	
Tested positive for COVID-19 (someone close)						F(4,3226)=3.66, p=0.006
Tested positive	14 (29.2%)	72 (32.9%)	140 (41.4%)	117 (22.1%)	923 (28.0%)	
Not tested or tested negative[a]	34 (70.8%)	147 (67.1%)	198 (58.6%)	412 (77.9%)	2377 (72.0%)	
COVID-19 health risk factors[b]	1.0 (1.2)	0.6 (1.1)	0.6 (1.2)	0.4 (0.9)	0.8 (1.1)	F(4,3226)=2,38, p=0.050
**Confidence determinants**						
Mistrust of vaccine benefit	2.9 (1.4)	2.9 (1.4)	2.6 (1.3)	2.6 (1.1)	2.3 (1.2)	F(4,4025)=7.41, p<0.001*[2,3]
Worries over unforeseen future effects	4.0 (1.3)	4.1 (1.3)	3.8 (1.3)	3.8 (1.1)	3.7 (1.3)	F(4,4025)=9.26, p<0.001*[2,3]
Concerns about commercial profiteering	3.5 (1.5)	3.7 (1.4)	3.4 (1.4)	3.2 (1.3)	2.9 (1.5)	F(4,4025)=9.44, p<0.001*[2,3]
Preference for natural immunity	3.7 (1.3)	3.5 (1.3)	3.4 (1.4)	3.3 (1.2)	3.2 (1.4)	F(4,4025)=2.20, p=0.067
Positive attitudes toward holistic health approaches	12.7 (5.0)	12.8 (5.4)	12.7 (5.1)	12.7 (4.1)	11.8 (4.2)	F(4,4025)=0.95, p=0.435
Positive attitudes toward complementary and alternative medicine	22.2 (4.3)	22.4 (4.3)	23.0 (4.2)	23.3 (3.9)	23.6 (5.0)	F(4,4025)=1.31, p=0.263
Mistrust in Government's management of COVID-19	23.8 (8.7)	26.0 (9.3)	26.1 (8.6)	25.3 (8.1)	26.0 (9.1)	F(4,4025)=0.36, p=0.836

[a]Reference variable.
[b]One point was assigned for each health risk factor (i.e., heart disease, hypertension, lung disease, diabetes, cancer, chronic kidney disease, obesity, and weakened immune system) to derive a total health risk factor score for COVID-19.
*p<0.003 (0.05/15 comparisons)

Bonferroni corrected p-value <0.05 with Whites as the reference group:
[1]Indigenous > White
[2]Black > White
[3]East Asian > White
[4]White > East Asian
